# Which is the best transcranial direct current stimulation protocol for migraine prevention? A systematic review and critical appraisal of randomized controlled trials

**DOI:** 10.1186/s10194-021-01361-0

**Published:** 2021-11-27

**Authors:** Raffaele Ornello, Valeria Caponnetto, Susanna Ratti, Giulia D’Aurizio, Chiara Rosignoli, Francesca Pistoia, Michele Ferrara, Simona Sacco, Aurora D’Atri

**Affiliations:** Neuroscience Section, Department of Applied Clinical and Biotechnological Sciences, University of L’Aquila, L’Aquila, Italy

**Keywords:** Transcranial direct current stimulation, migraine, non-pharmacological treatment, migraine prevention

## Abstract

**Background:**

Transcranial direct current stimulation (tDCS) could counteract the pathophysiological triggers of migraine attacks by modulating cortical excitability. Several pilot randomized controlled trials (RCTs) assessed the efficacy of tDCS for migraine prevention. We reviewed and summarized the state of the art of tDCS protocols for migraine prevention, discussing study results according to the stimulations parameters and patients’ populations.

**Main body:**

We combined the keywords ‘migraine’, ‘headache’, ‘transcranial direct current stimulation’, and ‘tDCS’ and searched Pubmed, Scopus, and Web of Science, from the beginning of indexing to June 22, 2021. We only included RCTs comparing the efficacy of active tDCS with sham tDCS to decrease migraine frequency, intensity, and/or acute drug utilization. The risk of bias of each RCT was assessed by using the RoB-2 tool (Cochrane Collaboration).

Thirteen RCTs (from 2011 to 2021) were included in the review. The included patients ranged from 13 to 135. RCTs included patients with any migraine (*n*=3), chronic migraine (*n*=6), episodic migraine (*n*=3) or menstrual migraine (*n*=1). Six RCTs used cathodal and five anodal tDCS, while two RCTs compared the efficacy of both cathodal and anodal tDCS with that of sham. In most of the cathodal stimulation trials, the target areas were the occipital regions, with reference on central or supraorbital areas. In anodal RCTs, the anode was usually placed above the motor cortical areas and the cathode on supraorbital areas. All RCTs adopted repeated sessions (from 5 to 28) at variable intervals, while the follow-up length spanned from 1 day up to 12 months. Efficacy results were variable but overall positive. According to the RoB-2 tool, only four of the 13 RCTs had a low risk of bias, while the others presented some concerns.

**Conclusions:**

Both anodal and cathodal tDCS are promising for migraine prevention. However, there is a need for larger and rigorous RCTs and standardized procedures. Additionally, the potential benefits and targeted neurostimulation protocols should be assessed for specific subgroups of patients.

**Supplementary Information:**

The online version contains supplementary material available at 10.1186/s10194-021-01361-0.

## Introduction

Migraine preventive treatments are indicated in case of high-frequency, highly disabling episodes, or scarce response to acute treatments. Pharmacological treatments of different classes, including antihypertensives, antidepressants, anticonvulsants, onabotulinumtoxinA, and treatments acting on the calcitonin gene-related peptide, are suitable for most patients with migraine [1]. Neuromodulation techniques are an appealing complement or alternative to pharmacological treatment. Neuromodulation can be used in patients who prefer non-pharmacological management or who cannot be adequately managed with pharmacological treatment [2].

Migraine has a complex and still unclear pathophysiology involving several circuits of both the central and peripheral nervous system [3, 4]. According to the most credited hypotheses, the activation of the trigeminovascular system, mostly pertaining to the peripheral nervous system, is the key event of migraine pain generation [5]; however, the central nervous system is also involved in the generation and perception of migraine [6]. The brain cortex of subjects with migraine is hyper-responsive to external stimuli [7], possibly due to altered functional connectivity with subcortical structures, including the thalamus [3]. The resulting “thalamo-cortical dysrhythmia” can be controlled by non-invasive neuromodulation techniques, including transcranial magnetic stimulation (TMS) and transcranial direct current stimulation (tDCS) [8]. tDCS is based on delivering a low-intensity current through the scalp by means of electrodes to modulate the state of polarization of the cerebral cortex; depending on the polarity of the electrical stimulation, tDCS can be either anodal or cathodal, whereas cathodal stimulation has a hyperpolarizing (inhibitory) effect and anodal stimulation has a depolarizing (excitatory) effect [9].

Several small randomized controlled trials (RCTs) showed the efficacy of different protocols of tDCS over sham stimulation for the prevention of migraine, as pointed out by previous systematic reviews and meta-analyses [10–13]. However, more and more high-quality RCTs of tDCS are being issued in recent years leading to a rapid evolution of the field. In those RCTs, the stimulation protocols widely differ, in the absence of any agreement. The International Headache Society published guidelines to improve and standardize the conduction of RCTs of neuromodulation for migraine prevention [14]; comparing those guidelines with the RCT methodology might help evaluating research in the field.

In the present review, we aimed at summarizing the knowledge we have so far on tDCS protocols for migraine prevention and providing insights and suggestions for the design of future trials.

## Methods

We conducted a systematic review according to the ‘Cochrane Handbook for Systematic Reviews of Interventions’, Version 5.1.0 [15]; to report results, we followed the ‘Preferred Reporting Items for Systematics Reviews and Meta-Analyses’ (PRISMA) guidelines which were updated in 2020 [16]. The review was submitted to PROSPERO on September 24^th^, 2021.

### Search strategy and study selection

We combined the keywords ‘migraine’, ‘headache’, ‘transcranial direct current stimulation’, and ‘tDCS’ and searched Pubmed, Scopus, and Web of Science on June 22nd, 2021. No additional filters were considered with the purpose of ensure high sensitivity of the search strategy. Retrieved references were managed with EndNote Free Web to remove duplicate publications.

Four raters (RO, VC, SR, GDA) selected the retrieved records in a two-step process, by first examining titles and abstracts and, subsequently, full texts of eligible references. In both steps, each record was assessed by two raters blinded to each other’s evaluation. Titles and abstracts were evaluated in full text if respecting the following criteria: a) to be primary studies (reviews, editorials, letters, case reports, and case series were excluded) with published data; b) to include migraine patients; c) to include a tDCS intervention. When the abstract was not available, the reference was considered eligible and evaluated in full text. To be included in the systematic review, full texts of eligible articles had to be RCTs of a tDCS intervention compared with sham intervention and to report migraine-related outcomes such as headache frequency or acute medications intake. In both steps of article selection, all records were evaluated by two raters. In case of disagreement, a discussion among all the raters was performed to reach an agreement.

### Data extraction and analysis

Four researchers (RO, VC, SR, GDA) used an electronic spreadsheet of Microsoft Excel for Windows to independently extract first author, publication year, number of included subjects, age and sex distribution, tDCS parameters (electrode size and position, current intensity, session length, number of sessions), assessment timepoints, and outcomes (headache/migraine frequency and intensity, acute drug consumption, other efficacy outcomes, adverse events). For each record, data were extracted by two raters blinded to each other’s assessment.

Due to the heterogeneity in tDCS montages and schedules, outcome definitions, and assessment timepoints, no formal meta-analysis was performed. The quality of the included RCTs was assessed by assessing their adherence to the guidelines of the International Headache Society guidelines [14]. The risk of bias of each RCT was assessed according to the RoB-2 tool proposed by the Cochrane Collaboration [17]. This assessment was performed on the outcomes of migraine frequency, severity, and acute treatment utilization.

## Results

### Literature search

**Fig. 1:**
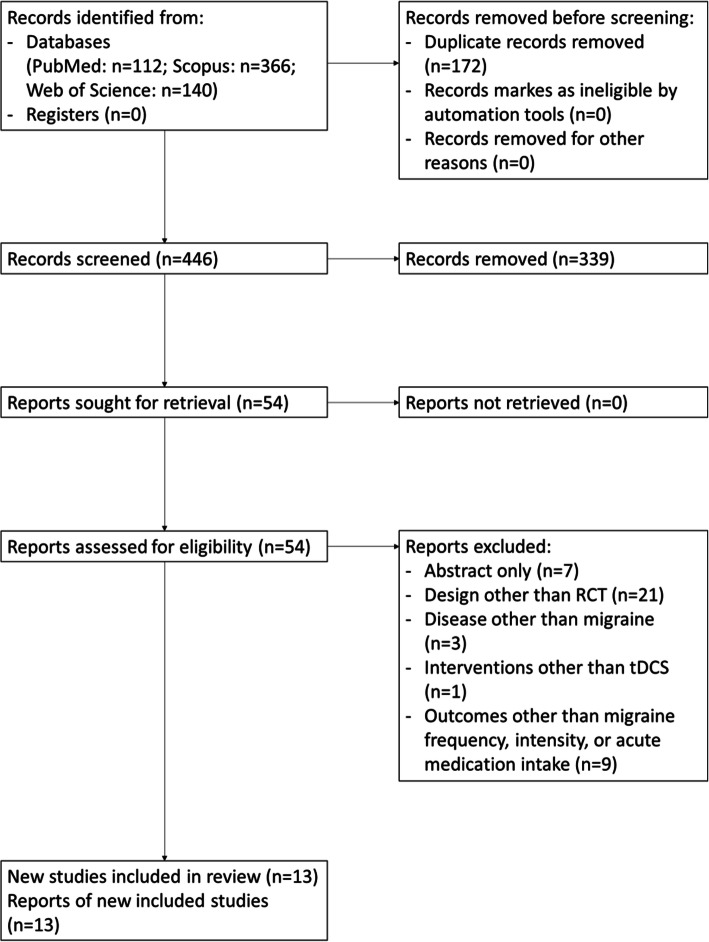
PRISMA 2020 flowchart of study selection

The database search identified 618 articles. After duplicate removal, 446 articles were screened for eligibility and 13 RCTs published from 2011 to 2021 were included in the review [18–30]. Details about study selection are reported in Fig. 1.

### Study populations

Details of the patient populations included in the 13 RCTs and of their tDCS parameters are reported in Table 1. The RCTs included 13 to 135 subjects; six trials included subjects with chronic migraine with or without medication overuse [19, 22–26], three included subjects with episodic migraine [18, 21, 27], and one only women with menstrual migraine [30], while the remaining three RCTs included subjects with any migraine [20, 28, 29]. Only two RCTs reported stratified results by the presence of aura [20, 27].

**Table 1:** Summary of randomized controlled trials of anodal transcranial direct current stimulation for migraine prevention

Study	N	Age range	Migraine type	Concomitant preventatives, %	Stimulation type	Electrode surface area (cm^**2**^)	Current intensity (mA)	Session duration (min)	Baseline observation (before the first tDCS session)	Follow-up (after the last tDCS session)
**Ahdab, 2019** **[**18**]**	42 (cross-over)	18-60	Episodic	7	Cathodal	35	2	20	1 week	2 weeks
**Andrade, 2017** **[**19**]**	6 active (M1), 3 active (DLPFC), 4 (sham)	18-65	Chronic	NR	Anodal	25	2	20	1 day	1 day
**Antal, 2011** **[**20**]**	13 active, 13 sham	20-53	Episodic/chronic	0	Cathodal	35	1	15	8 weeks	8 weeks
**Auvichapayat, 2012** **[**21**]**	20 active, 17 sham	18-65	Episodic	0	Anodal	35	2	20	4 weeks	12 weeks
**Dalla Volta, 2020** **[**22**]**	28 active, 17 sham	18-65	Chronic, without aura	100 (topiramate)	Cathodal	35	1.5	15	NR	120 days
**DaSilva, 2012** **[**23**]**	8 active, 5 sham	18-60	Chronic	NR	Anodal	35	2	20	1 week	120 days
**De Icco, 2021** **[**24**]**	10 active, 10 sham	18-65	Chronic (with medication overuse)	0	Anodal	Anode: 9 Cathode: 24	2	20	1 month	6 months
**Grazzi, 2020** **[**25**]**	44 active (anodal), 45 active (cathodal), 46 sham	18-65	Chronic (with medication overuse)	NR	Anodal/cathodal	35	2	20	1 month	12 months
**Mansour, 2019** **[**26**]**	18 (cross-over)	18-60	Medication overuse	28	Anodal/cathodal	35	2	20	1 week	2 weeks
**Pohl, 2020** **[**27**]**	11 active, 12 sham	18-80	Episodic	NR	Anodal	Anode: 35 Cathode: 100	1	20	4 weeks	4 months
**Rahimi, 2020** **[**28**]**	15 active (M1), 15 active (S1), 15 sham	18-60	Episodic/chronic	0	Cathodal	Cathode: 15 Anode: 35	1	20	4 weeks	12 months
**Rocha, 2015** **[**29**]**	10 active, 5 sham	18-50	Episodic/chronic	0	Cathodal	35	2	20	30 days	30 days
**Wickmann, 2015** **[**30**]**	8 active, 8 sham	NR	Menstrual	0	Cathodal	35	2	20	12 weeks	12 weeks

*DLPFC* indicates dorsolateral prefrontal cortex, *M1* primary motor area, *NR* not reported, *S1* primary somatosensory area

### tDCS technical parameters, schedules, and assessment timepoints

**Fig. 2:**
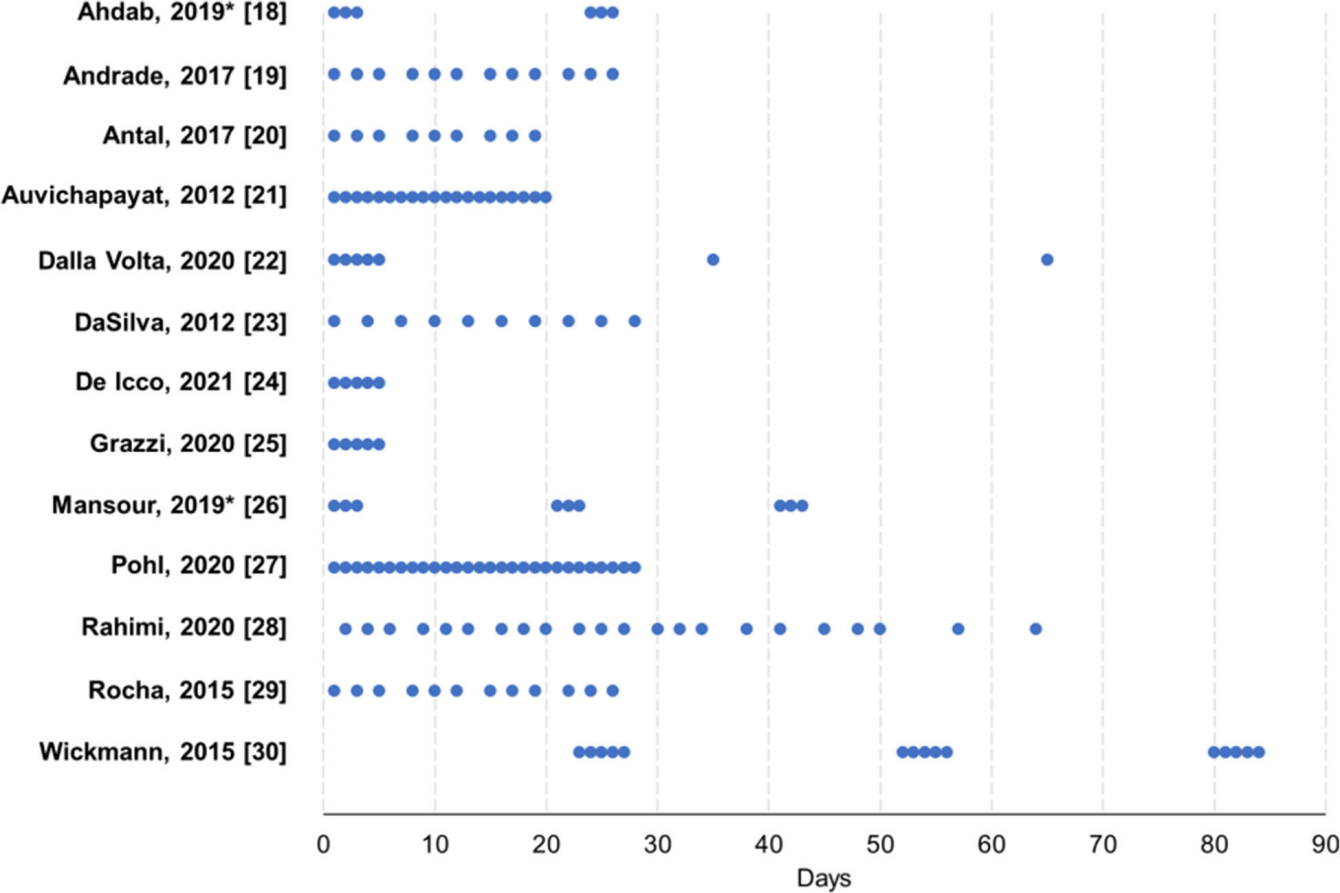
Overview of transcranial direct current stimulation schedules in the included randomized controlled trials. Each dot represents a single session. *cross-over trial; each group of dots represents a different intervention

In most RCTs, the electrode surface area was 35 cm^2^, current intensity was 2 mA, and the duration of each tDCS session was 20 minutes (Table 1). The number of sessions ranged from 5 to 28, at variable distance between each other, while the length of follow-up ranged from 1 day to 12 months after the last tDCS session. Figure 2 reports details about the tDCS stimulation schedules.

### tDCS montages

**Fig. 3:**
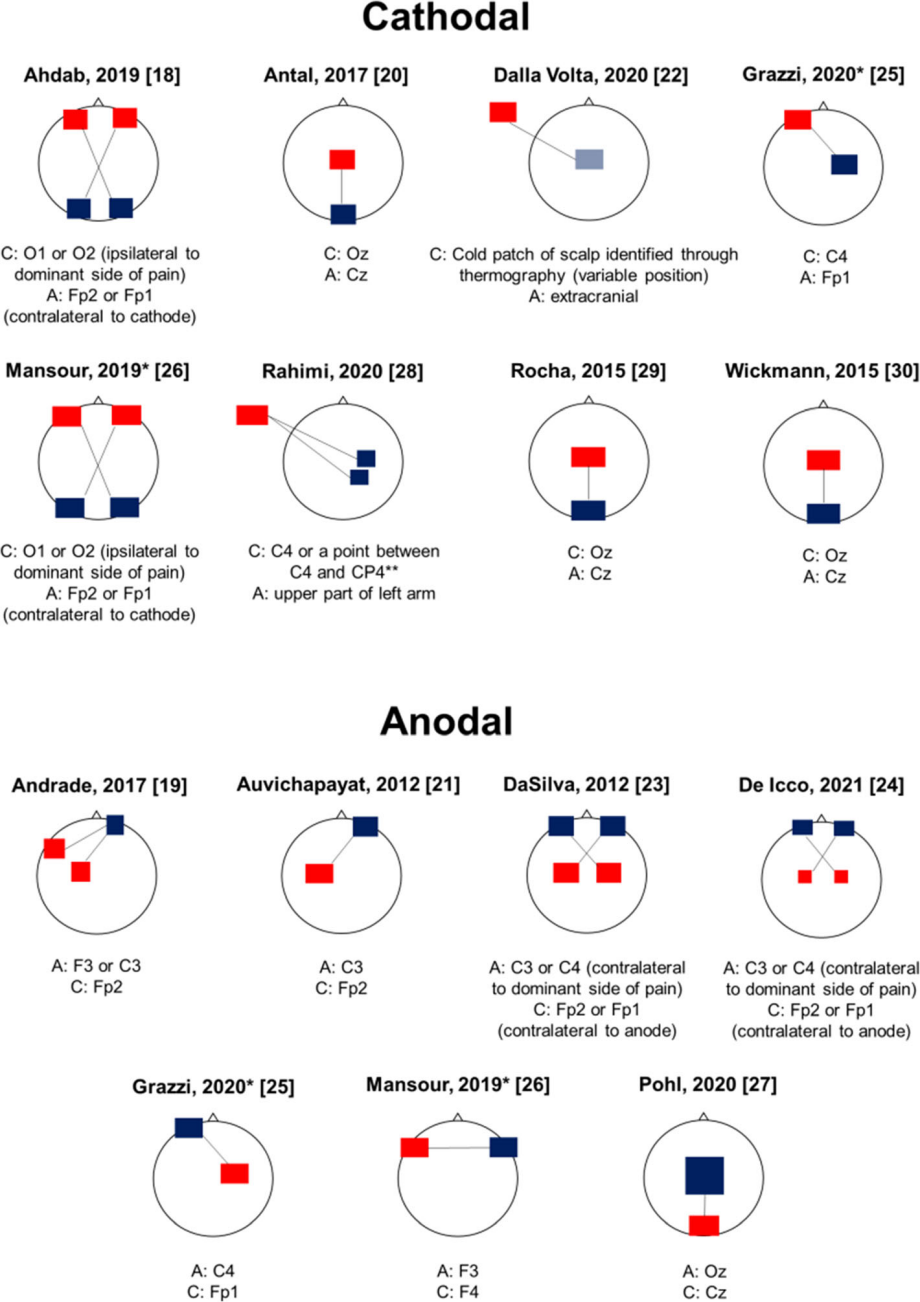
Overview of transcranial direct current stimulation montages in the included randomized controlled trials. Blue squares/rectangles indicate cathodes, while red squares/rectangles indicate anodes. Electrodes’ shape and dimension reflect the differences in RCTs stimulation setups. Solid lines connect the electrodes pairs that have been tested in different stimulation conditions in each RCT. The positions of anodes and cathodes are identified according to the international 10/20 electroencephalographic system. *This study assessed both cathodal and anodal stimulation. **10/10 electroencephalographic system

Six RCTs assessed the efficacy of cathodal tDCS [18, 20, 22, 28–30], five assessed the efficacy of anodal tDCS [19, 21, 23, 24, 27], and two of both techniques [25, 26]. Figure 3 reports the montages used for tDCS in each RCT.

#### Cathodal tDCS

In three RCTs, the cathode was positioned occipitally (Oz electrode according to the 10/20 international EEG system), with central reference (Cz electrode) [20, 29, 30]; in two trials, the cathode was positioned occipitally ipsilateral to the dominant side of migraine (O1 or O2 electrode), with contralateral supraorbital reference (Fp2 or Fp1 electrodes) [18, 26]; in one trial, the cathode was placed on the right M1 (C4 electrode), with left supraorbital reference (Fp1 electrode) [25]; in a further trial, the cathode was positioned above the right M1 (C4 electrode) or primary sensory area (S1; CP4 electrode in the 10/10 EEG system) with extracranial reference [28]; in the remaining RCT, the position of the cathode was variable and determined by thermography, with the identification of a “cold patch” over the scalp, with extracranial reference [22].

#### Anodal tDCS

In four RCTs of anodal tDCS, the anode was positioned above M1, with varying sides (C3 or C4 electrodes), with contralateral supraorbital reference (Fp2 or Fp1 electrodes) [21, 23, 24, 31]; one RCT placed anodes either above the left M1 or above the left dorsolateral prefrontal cortex (DLPFC; C3 or F3 electrodes, respectively), with contralateral supraorbital reference (Fp2) [19]; one RCT placed the anode over the left DLPFC (F3 electrode), with reference on the opposite side (F4 electrode) [26]; a further trial positioned the anode on the right M1 (C4 electrode) with left supraorbital reference (Fp1 electrode) [25]; the remaining RCT placed the anode above the occipital areas (Oz electrode) with central reference (Cz electrode) [27].

### Adherence to guideline standards

**Fig. 4:**
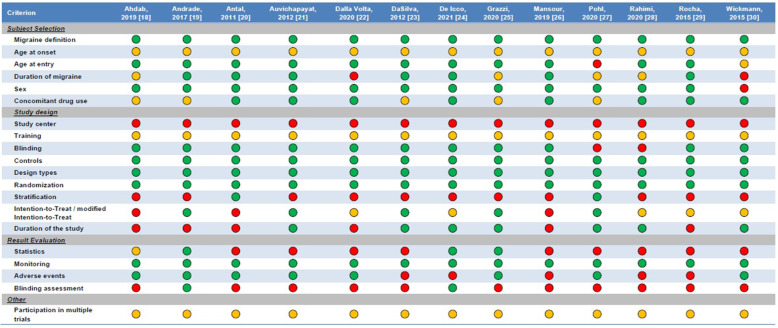
Adherence of available randomized controlled trials on transcranial direct current stimulation to the guideline recommendations for clinical trials of neuromodulation devices for the treatment of migraine in adults [14]. A green circle indicates that the criterion was met; a red circle, that it was not met; an amber circle, that it was not specified

Figure 4 displays the characteristics of the included RCTs compared with those required by international guidelines [14]. All RCTs provided an adequate definition of migraine, had an adequate trial design, and reliably monitored patients’ outcomes by using headache diaries. Of note, none of the RCTs was multicenter. Five RCTs did not report details about the concomitant preventive treatments for migraine taken by patients [18, 19, 23, 25, 27], while only two RCTs performed an assessment of blinding [19, 24].

The outcomes reported by the included RCTs mostly referred to migraine or headache days, headache intensity, and acute treatment utilization; questionnaires to assess patient-reported outcomes, such as disability and quality of life, were only used by few RCTs; no RCT assessed the usability of tDCS devices or quantified their costs (Figure 4).

### Efficacy and safety

**Fig. 5:**
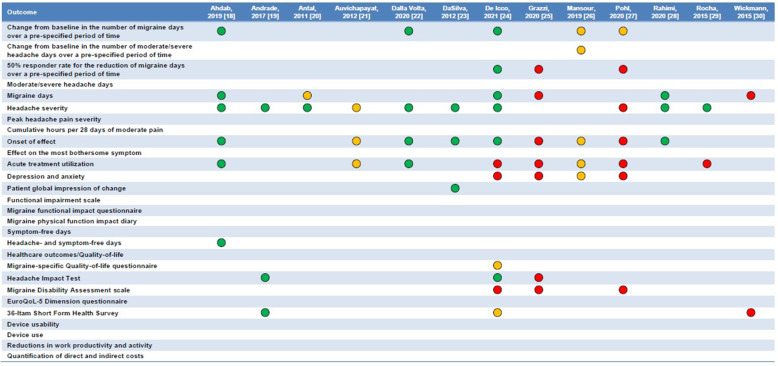
Results of the available randomized controlled trials comparing transcranial direct current stimulation to sham procedure. The list is taken from the guidelines of clinical trials of neuromodulation devices for the treatment of migraine in adults [14]. More detailed quantitative reports are provided in Supplemental file 1. Green circles indicate that tDCS was entirely superior to sham, amber circles that tDCS was partially superior to sham, red circles that tDCS was note superior to sham; cells were left empty if outcomes were not reported

**Fig. 6:**
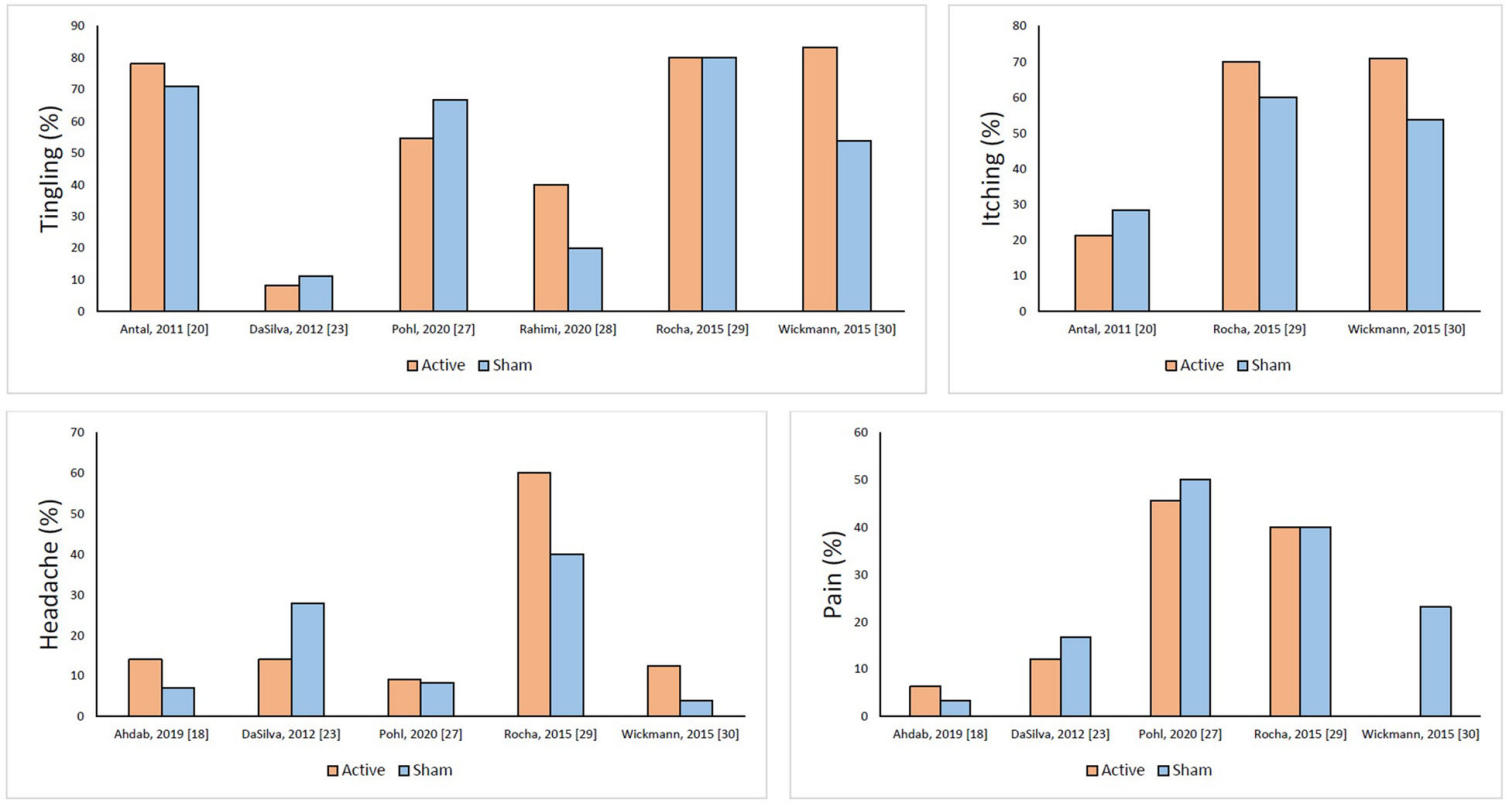
Proportion of patients with tingling, itching, headache, and pain during tDCS in the included randomized controlled trials. No difference between active and sham groups was significant

A schematic representation of the efficacy data of included RCTs is shown in Figure 5, while detailed efficacy data are reported in Supplementary file S1. Most RCTs showed a significantly greater decrease in headache frequency, intensity, and acute medication consumption in the active groups compared with sham. Results did not show any difference between active tDCS and sham in the RCT of anodal stimulation over the occipital areas [27], in which only headache intensity decreased in the active group compared with sham, in the trial on patients with medication overuse [24], and in the RCT of tDCS in menstrual migraine [30]. The presence of aura did not influence outcomes in one RCT [27], while in another RCT patients with migraine with aura had a higher advantage than those without aura in terms of headache intensity reduction [20]. In one RCT the effect of tDCS waned after 12 weeks [21], while in another RCT the effect was detectable up to 6 months after a 5-day intervention [24]. In all RCTs, adverse events mostly occurred during the tDCS procedure and were mild and transient. Figure 6 reports the difference between active and sham groups in the four most reported adverse events, i.e., tingling, itching, headache, and pain; more details are provided in Supplementary file S2. Notably, none of the RCTs found differences in adverse events between active and sham groups. No serious adverse event was reported by any RCT.

### Risk of bias

Details of the risk of bias assessment are reported in Supplementary file S3. Only four of the 13 RCTs included had a low risk of bias [19, 23–25], while the remaining nine RCTs presented some concerns [18, 20–22, 26–30]; no study had a high risk of bias.

## Discussion

Our systematic review showed that either cathodal or anodal tDCS can have some benefit for the prevention of migraine; however, the available RCTs are mostly pilot studies not fulfilling all the quality criteria. Overall, the technique proved to be beneficial for the decrease in migraine frequency, intensity, and acute drug utilization; however, the tDCS schedules and montages used in the RCTs were heterogeneous. Hence, it is not possible to date to recommend any tDCS approach that best suits patients with migraine.

It is important to promote consensus over tDCS protocols to spread the use of this technique for clinical purposes in patients with migraine. For this reason, the primary aim of this study was to provide a systematic review focused on methodological criteria of the available studies, and particularly on stimulation protocols and patient selection. Across the available RCTs, many factors of heterogeneity should be considered, referring to patient population, stimulation protocols, assessment timepoints, and outcomes.

### Patients’ characteristics

The available RCTs included either subjects with episodic [18, 21, 27] or with chronic migraine [19, 22, 23], while some trials selected subjects with medication overuse [24–26]; three additional RCTs included subjects with both episodic and chronic migraine [20, 28–30]. In all those populations, tDCS led to some therapeutic effect. However, the RCTs were overall underpowered to allow any subgroup analysis. Only two RCTs performed exploratory subgroup analyses in patients with and without aura, showing similar efficacy in the two subgroups [20, 27].

The available RCTs either excluded patients with concomitant migraine preventive medication [20, 21, 24, 26, 28–30] or did not report the proportion of patients treated with preventive drugs [19, 23, 25, 27]; only in one RCT tDCS was used in combination with topiramate in all patients [22]. Even in the RCT on patients using topiramate, tDCS showed some efficacy compared with sham. Besides, in two RCTs tDCS enhanced the effect of detoxication for patients with medication overuse [24, 26]. According to guideline recommendations, the inclusion in neuromodulation RCTs of patients with concomitant preventive medication should be liberal, provided that patients do not change their medication for at least 3 months [14]. In the absence of better data, tDCS can be considered suitable for patients with migraine irrespective of their concomitant treatments, even if most of the evidence is available for patients without concomitant treatments.

A further point to consider for patient selection are prior preventive treatment failures. Considering those failures has become increasingly important in recent years due to the eligibility criteria for treatments acting on the calcitonin gene-related peptide [32, 33]. Most RCTs did not report the proportion of patients who had failed preventive treatments for their migraine; one RCT only included patients who had not tried any preventive treatment [21], while one further RCT only included patients with three or more failures [24]. Our review shows that there are currently no available data to conclude whether the efficacy of tDCS changes according to previous preventive treatment failures. In our opinion, tDCS can be best offered to patients with frequent and severe migraine who are resistant or refractory to preventive medication [2]. However, the technique might also be an option for patients who refuse drugs or with contraindications to migraine preventive treatments.

### Stimulation schedules and assessment timepoints

The RCTs included in the present systematic review adopted different stimulation schedules (Figure 2); there is no direct comparison between different protocols, leaving uncertainty about the optimal stimulation protocol over time. In clinical practice, the number of possible consecutive tDCS sessions is limited by the fact that tDCS is performed by trained personnel during working hours. Self-administered tDCS would obviate to this problem. However, the results of the only RCT assessing the efficacy of self-administered tDCS [27] were worse than those of other RCTs (Figure 5), suggesting that self-administered tDCS should still be tested.

A question that may arise in clinical practice is about the duration of tDCS effect, in order to plan repetitive stimulation. The duration of effect of tDCS was noted up to 12 months in one RCT [25]; however, some other trials showed a waning effect of tDCS over 3-4 months [18, 21]. Notably, the RCT with the worst efficacy results was also the one with the longest follow-up [25]. The duration of tDCS effect might be influenced by the stimulation schedule. However, it is difficult to estimate the number of consecutive sessions that ensure the longest duration of tDCS effect, as each of the available RCTs adopted a different approach.

### Trial methodology

Overall, the quality of RCTs of tDCS for migraine prevention is not high. Only few of the outcomes recommended by international guidelines [14] were reported by RCTs (Figure 4). RCTs mostly focused on headache intensity and frequency; outcomes that are often reported by pharmacological RCTs, such as the proportion of patients with a ≥50% decrease in migraine days compared with baseline, were seldom reported (Figure 4). International guidelines recommend reporting patient-reported outcomes, together with outcomes related to device usability and costs [14], which were not reported by the RCTs of tDCS. There is a need for a more thorough assessment of migraine-related outcomes in neuromodulation trials, to better compare their efficacy with that of drugs and integrate those non-pharmacological treatments into clinical practice guidelines.

Most of the included RCTs had some methodological concerns according to the Cochrane RoB-2 tool; only four RCT had a low risk of bias (Supplemental file S1). Some concerns in the risk of bias were detected in most assessment domains. Several RCTs were conceived as pilot trials, while there is a need for more complete RCTs compliant with the most recent guidelines.

### Stimulation sites and neuroanatomical targets

To date, the preferable target of tDCS for migraine prevention still has to be determined. Cathodal, i.e., inhibitory, tDCS mostly targeted the occipital cortex (Figure 3). The visual cortex is hyper-responsive in patients with migraine compared with non-migraineurs, as shown by experimental evidence [34, 35]. Therefore, the rationale of hyperpolarizing the visual cortex of patients with migraine by cathodal electrical stimulation is to avoid the hyper-responsivity of the visual cortex. On the other hand, RCTs performing anodal (excitatory) tDCS targeted the M1 or DLPFC (Figure 3) with the aim of inducing a reflex inhibition of subcortical pain-generating areas, centered on the thalamus; this approach has been already adopted in RCTs of tDCS for fibromyalgia and other chronic pain conditions [36]. More specifically, the stimulation of the M1 causes a direct inhibition of the thalamus [37], while excitatory stimulation of the DLPFC can inhibit the midbrain-medial thalamic pathway [38].

The available literature cannot suggest any tDCS montage that is better than others at preventing migraine. Each montage targets specific neural structures, all potentially implied in migraine pathogenesis [36]. Preliminary identification of the neuroanatomical targets most suitable to each patient would be an important advance for a wider employment and better results of tDCS.

### The role of functional tests

In our opinion, a first step toward the identification of adequate targets for tDCS is studying the effect of this intervention with functional tests; however, those tests were rarely used by the available RCTs and highly heterogenous in nature. Referring to the three RCTs included in the present review, one verified the neurophysiologic effect of tDCS by using EEG [24], the second with the detection of phosphene threshold [29], and the third with a computational model based upon functional neuroimaging [23]. At EEG, anodal tDCS of the primary motor cortex resulted in increased alpha power over the occipital cortex; this effect could be attributed to either direct modulation of cortical activity or to the decrease of the thalamo-cortical dysrhythmia observed in migraineurs [24]. Compared with EEG, results related to phosphene threshold were more controversial. Phosphene threshold is lower in migraineurs compared with non-migraineurs due to higher responsiveness of visual cortex [39]. The RCT included in the present review confirmed that phosphene threshold, assessed through transcranial magnetic stimulation, was lower in migraineurs than in non-migraineurs; however, occipital cathodal tDCS was not able to increase that threshold, suggesting that cortical hyper-responsiveness persisted in migraineurs [29]. In another RCT, a computational model showed that anodal tDCS over the motor cortex was able to generate electrical fields not only in the cortex, but also in the insula, cingulate cortex, thalamus, and brainstem, all of which contain circuits implied in the genesis of migraine [23].

Functional tests could be useful not only to monitor the effects of tDCS, but also to select patients who might respond to the treatment. In one RCT a neurophysiologic test, i.e., low phosphene threshold of the visual cortex, was adopted as selection criteria for cathodal tDCS [29]. An open-label study [40] that was not included in the present review due to its design also showed the effectiveness of cathodal tDCS over the visual cortex in selected patients with abnormal excitability of the visual cortex. A further RCT selected a “cold patch” of the head by thermography as the target of cathodal tDCS [22], as previous evidence showed that those cold patches could be related to migraine generation; however, there was no replication of this technique in further RCTs. Functional tests could help improving not only the efficacy of neuromodulation interventions, but also our understanding of the pathophysiology of migraine.

### Is the presence of aura a selection criterion for tDCS?

A further element that could determine the response of migraine to neuromodulation is the presence of aura. Aura is assumed to be the clinical correlate of cortical spreading depression (CSD), a spreading wave of depolarization that is followed by a decrease in neuronal activity and blood flow [41–43]. There is indirect experimental evidence that CSD is linked to aura in patients with migraine [44, 45]. Therefore, the activation of brain cortex could be a prominent phenomenon in migraine with aura, while in migraine without aura subcortical structures, including the thalamus, hypothalamus, and brainstem can play a prominent role [4, 46]. Evidence suggests that neuromodulation techniques such as transcranial magnetic stimulation might act better on migraine with aura than on migraine without aura [47, 48]. Surprisingly, tDCS had similar effect sizes in both migraine with and without aura in two RCTs [20, 27]. This lack of difference might depend on the low number of patients included in RCTs, too low to allow the detection of significant effects. As a further point, patients might have low-frequency auras, therefore hindering a specific effect of tDCS on aura.

### Suggestions for future research

**Fig. 7:**
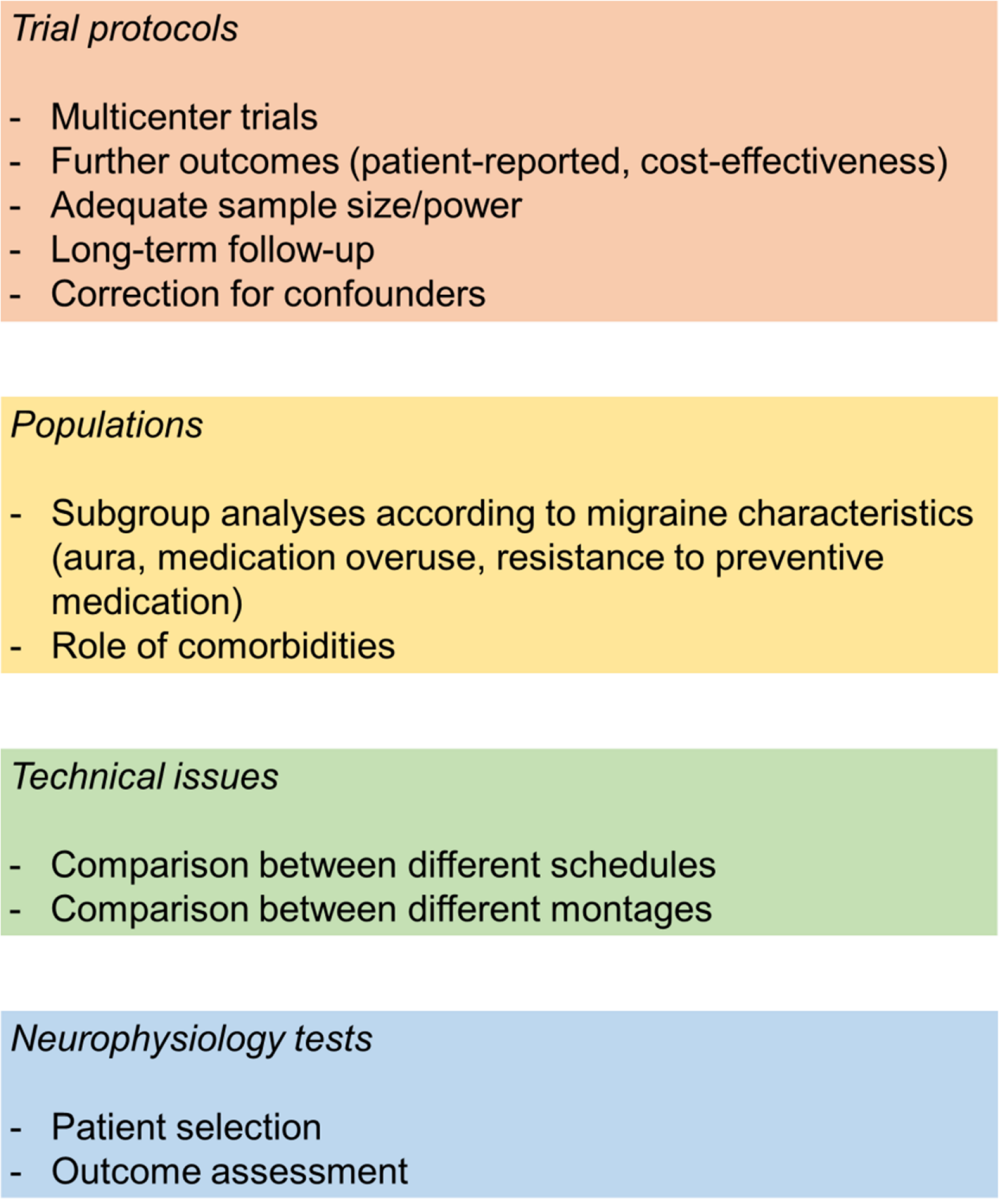
Unmet needs in transcranial direct current stimulation trials for migraine prevention

According to the results of the available trials, it is not possible to determine which approach is the best to prevent migraine. Nevertheless, the positive results of most RCTs encourage new studies to provide conclusive evidence. The available RCTs present some issues that could be solved by further studies; the unmet needs of tDCS research in the field of migraine are summarized in Figure 7. Referring to methods, there is a need for multicenter RCTs with adequate sample size calculations, strategies to control for confounding factors and incomplete data, and to perform subgroup analyses for the different populations of interest. The range of reported outcomes should also be expanded to include patient-reported outcomes and cost-effectiveness assessments, as suggested by international guidelines [14]. Referring to tDCS schedules, RCTs suggested a precise range of current intensity (1 to 2 mA) and duration of each session (15-20 minutes); however, there is no consensus over the optimal number of sessions and time interval between them. Future RCTs should compare different schedules and monitor the onset of tDCS effect and its duration over time. Referring to tDCS montages, excitatory stimulation is best centered over motor areas, while inhibitory stimulation is best centered over the visual cortex. Because both the cathode and anode of tDCS can perform a stimulation, positioning the anode (excitatory stimulation) over the motor cortex and the cathode (inhibitory stimulation) over the visual cortex might stimulate at once most of the brain areas that are relevant for migraine prevention. The help of neurophysiological tests to select appropriate patient groups and montages, as well as to monitor tDCS outcomes, could largely improve research in the field and consequently clinical practice.

### Strengths and limitations

The present review systematically reported the results of the available RCTs on tDCS for migraine prevention and is a substantial update to previous reviews [10–13], as several important RCTs were published in the last two years. We chose to maintain a conservative approach by not performing a meta-analysis, given the remarkable heterogeneity across RCTs. Despite those strengths, the inclusion of RCTs only could constitute a limitation, as observational studies could have added important information to the topic. Besides, the heterogeneity of RCTs did not allow to draw definite conclusions on the best possible tDCS protocol for the prevention of migraine.

## Conclusions

tDCS is a non-invasive neuromodulation technique highly promising to effectively prevent migraine without relevant harms to patients. An advantage of tDCS is its non-pharmacological nature, which makes it suitable for patients with comorbidities or poor tolerance to pharmacological treatments. Besides, tDCS acts on the central nervous system thus potentially being synergic with migraine preventive drugs that mostly act at a peripheral level. However, there is still an unmet need of high-quality RCTs supporting a wider use of tDCS and a need for standardization of the procedure.

## Supplementary Information

**Additional file 1.** Summary of results of included trials.

**Additional file 2.** Adverse events in the included trials.

**Additional file 3.** Risk of bias analysis.

## Data Availability

Data sharing is not applicable to this article as no datasets were generated or analysed during the current study.
