# Supplementary material for: Which is the best transcranial direct current stimulation protocol for migraine prevention? A systematic review and critical appraisal of randomized controlled trials
Source: J Headache Pain. 2021 Nov 27;22(1):144. doi: 10.1186/s10194-021-01361-0 (PMC8903540; doi:10.1186/s10194-021-01361-0)
Supplement: Supplementary file 1 — Additional file 1. Summary of results of included trials. [file 10194_2021_1361_MOESM1_ESM.docx]

Summary of results of included trials

| **Outcome** | **Study** | **Results in active group** | **Results in sham group** | **Difference between active and sham** |
| --- | --- | --- | --- | --- |
| Change from baseline in the number of migraine days over a pre-specified period of time | Ahdab, 2019 [18] | -1.60±0.23 days at week 1  -1.50±0.26 days at week 2 | NR | NR |
|  | Dalla Volta, 2020 [22] | −42.7±65.4% | −11.3±18.0% | p=0.016 |
|  | De Icco, 2021 [24] | -11.6 days at month 1 -8.6 days at month 2 | -6.7 days at month 1 -4.5 days at month 2 | P=0.016 |
|  | Mansour, 2019 [26] | Effect size for prefrontal stimulation:  -2.00 (95% CI -3.51 to -0.49) at week 1 -1.06 (95% CI -2.56 to +0.45) at week 2  Effect size for occipital stimulation:  -2.78 (95% CI -4.28 to -1.27) at week 1 -2.00 (95% CI -3.51 to -0.49) at week 2 | NR | Significant difference at week 1 but not at week 2 for prefrontal tDCS; significant difference at week 1 and week 2 for occipital tDCS |
|  | Pohl, 2020 [27] | −1.7±0.5 monthly migraine days at week 16 | +0.2±0.4 monthly migraine days at week 16 | p=0.010 |
| Change from baseline in the number of moderate/severe headache days over a pre-specified period of time | Mansour, 2019 [26] | Effect size for prefrontal stimulation: -1.72 at week 1 NR at week 2  Effect size for occipital stimulation: -2.44 at week 1 -1.61 at week 2 | NR | Significant difference at week 1 (p=0.001) and week 2 (p=0.029) for occipital stimulation; significant interaction with time at week 1 only for prefrontal stimulation |
| 50% responder rate for the reduction of migraine days over a pre-specified period of time | De Icco, 2021 [24] | 70% at month 1 50% at month 2 | 20% at month 1 0% at month 2 | p=0.010 |
|  | Grazzi, 2020 [25] | 64.1% (anodal), 60.0% (cathodal) at month 12 | 46.3% at month 12 | No difference |
|  | Pohl, 2020 [27] | 36.3% at week 12 | 16.7% at week 12 | No difference |
| Migraine days | Ahdab, 2019 [18] | 3.3* (weekly) at baseline  1.8* at week 1  1.9* at week 2 | 2.5* (weekly) at baseline  2.0* at week 1  1.9* at week 2 | NR |
|  | Antal, 2011 [20] | 16.2±2.4 (monthly) at baseline 9.31±1.5 at week 8 | 12.8±2.77 (monthly) at baseline 11.0±3.5 at week 8 | No difference |
|  | De Icco, 2021 [24] | 15.8±3.1 (monthly) at baseline 4.6±3.3 at month 1  7.2±5.8 at month 2 | 15.7±2.3 (monthly) at baseline 9.5±4.2 at month 1 11.2±3.5 at month 2 | P=0.016 |
|  | Grazzi, 2020 [25] | Anodal:  20.4±6.6 (monthly) at baseline 13.3±10.2 at month 6 11.0±7.5 at month 12  Cathodal: 23.5±7.0 at baseline 11.8±8.6 at month 6 11.5±8.6 at month 12 | 21.8±6.4 (monthly) at baseline 12.6±8.9 at month 6 13.0±7.8 at month 12 | No difference |
|  | Rahimi, 2020 [28] | Motor cortex stimulation:  17.13±10.049 (monthly) at baseline 1.80±1.082 immediately after tDCS 2.93±1.53 at month 12  Sensory cortex stimulation: 14.00±8.61 at baseline 1.67±1.17 immediately after tDCS 2.20±1.93 at month 12 | 13.13±9.13 at baseline 10.27±7.73 immediately after treatment 10.93±7.076 at month 12 | NR |
|  | Wickmann, 2015 [30] | 4.62±1.6 days during 12-week stimulation | 6.12±0.8 days during 12-week stimulation | No difference |
| Headache severity | Ahdab, 2019 [18] | 2.4* points at baseline  1.3* at week 1  1.4* at week 2  Significant decrease at week 1 (-1.17±0.17) and week 2 (-1.01±0.19) compared with baseline | 2.0* points at baseline  1.5* at week 1  1.7* at week 2  No significant decrease | NR |
|  | Andrade, 2017 [19] | M1 stimulation: 7.1 (median) at baseline 4.3 immediately after tDCS  DLPFC stimulation: 8.0 at baseline 2.2 immediately after | 6.7 (median) at baseline  6.3 immediately after | NR |
|  | Antal, 2011 [20] | Mean VAS score:  1.99±0.1 at baseline 1.54±0.2 at week 8 | Mean VAS score: 1.88±0.1 at baseline 1.92±0.2 at week 8 | p=0.05 |
|  | Auvichapayat, 2012 [21] | Reduction in pain intensity: 1.50 (95% CI 1.18 to 1.82) at week 4 1.30 (95% CI 1.03 to 1.57) at week 8 0.35 (95% CI -0.07 to 0.62) at week 12 | Reduction in pain intensity: 0.53 (95% CI: 0.26 to 0.79) at week 4 0.18 (95% CI -0.03 to 0.38) at week 8 -0.12 (95% CI -0.29 to 0.05) at week 12 | NR |
|  | Dalla Volta, 2020 [22] | −31.1±36.9% | 8.3±13.5% | p=0.004 |
|  | DaSilva, 2012 [23] | 4.6±2.1 at baseline 4.7±2.7 at day 15 3.7±2.0 at day 30 3.1±2.7 at day 60 2.9±2.9 at day 120 | No change | NR |
|  | De Icco, 2021 [24] | F_1,18_=4.485; p=0.048 | F_1,18_=8.449; p=0.009 | NR |
|  | Pohl, 2020 [27] | −0.3±0.2 | 0.0±0.2 | No difference |
|  | Rahimi, 2020 [28] | Motor cortex stimulation:  7.67±1.71 at baseline 1.40±0.910 immediately after tDCS 2.00±1.36 at month 12  Sensory cortex stimulation: 6.47±1.64 at baseline 0.93±0.704 immediately after tDCS 1.27±0.704 at month 12 | 7.60±2.13 at baseline 7.00±1.89 immediately after treatment 7.13±1.85 at month 12 | NR |
|  | Rocha, 2015 [29] | No decrease during or after stimulation | No decrease during or after stimulation | No difference |
| Onset of effect | Ahdab, 2019 [18] | Week 1 | No effect | Not applicable |
|  | Auvichapayat, 2012 [21] | Week 4 | Week 4 | Not applicable |
|  | Dalla Volta, 2020 [22] | 10 days | No effect | Not applicable |
|  | DaSilva, 2012 [23] | 30 days | No effect | Not applicable |
|  | De Icco, 2012 [24] | 1 month | No effect | Not applicable |
|  | Grazzi, 2020 [25] | No effect | No effect | Not applicable |
|  | Mansour, 2019 [26] | Week 1 | No effect | Not applicable |
|  | Pohl, 2020 [27] | Week 8 | No effect | Not applicable |
|  | Rahimi, 2020 [28] | Immediately after tDCS | No effect | Not applicable |
| Acute treatment utilization | Ahdab, 2019 [18] | 0.57* at baseline  0.26* at week 1  0.27* at week 2  Significant decrease in tablet consumption at week 1 (-0.30±0.06) and week 2 (-0.22±0.08) compared with baseline | 0.42* at baseline  0.37* at week 1  0.43* at week 2  No significant decrease compared with baseline | NR |
|  | Auvichapayat, 2012 [21] | Reduction in mean abortive medications from baseline: 5.40 (95% CI 4.42 to 8.89) at week 4 2.58 (95% CI 2.22 to 7.18) at week 8 1.70 (95% CI 0.31 to 5.59) at week 12 | Reduction in mean abortive medications from baseline: 3.85 (95% CI 2.73 to 4.92) at week 4 1.83 (95% CI 1.05 to 2.60) at week 8 1.0 (95% CI -0.29 to 0.05) at week 12 | NR |
|  | Dalla Volta, 2020 [22] | −54.3±37.4% | −16.0±19.6% | P<0.0001 |
|  | De Icco, 2012 [24] | 31.8±13.4 (monthly doses) at baseline 9.1±4.7 at month 1 10.2±7.1 at month 2 | 35.4±11.9 (monthly doses) at baseline 10.0±4.0 at month 1 14.6±7.9 at month 2 | No difference |
|  | Grazzi, 2020 [25] | Anodal stimulation: 20.9±6.2 (monthly doses) at baseline 11.4±10.2 at month 6 10.6±8.4 at month 12 Cathodal stimulation:  23.1±6.9 at baseline 10.9±9.6 at month 6 10.4±10.4 at month 12 | 21.5±6.9 (monthly doses) at baseline 12.7±11.6 at month 6 12.6±8.9 at month 12 | No difference |
|  | Mansour, 2019 [26] | Prefrontal stimulation: 2.25±0.51 pills per day at baseline 1.48±0.32 at week 1 1.56±0.37 at week 2  Occipital stimulation: 2.56±0.54 pills per day at baseline 1.57±0.36 at week 1 1.36±0.35 at week 2 | 2.00±0.44 pills per day at baseline 2.17±0.42 at week 1 2.06±0.44 at week 2 | NR |
|  | Pohl, 2020 [27] | Change in monthly medication days: −0.9±0.4 at week 16 | Change in monthly medication days: −1.0±0.4 at week 16 | No difference |
|  | Rocha, 2015 [29] | Reduction in painkiller intake | No reduction in painkiller intake | Difference in favor of active group |
| Depression and anxiety | Grazzi, 2020 [25] | STAIS score change from baseline to month 12:  -2.3±13.1 points (anodal) -1.5±12.2 points (cathodal)  STAIT score change from baseline to month 12: -6.8±9.8 points (anodal) -3.0±9.2 points (cathodal)  BDI score change from baseline to month 12: -6.6±7.4 points (anodal) -4.7±8.1 points (cathodal) | STAIS score change from baseline to month 12: -4.4±9.0 points  STAIT score change from baseline to month 12: -4.2 12.0  BDI score change from baseline to month 12: -5.0 7.5 | No difference |
|  | De Icco, 2021 [24] | No modification in HADS score | No modification in HADS score | No difference |
|  | Pohl, 2020 [27] | Mean change in HADS-A score: -0.6±0.7 at day 50 -1.8±1.7 at day 218  Mean change in HADS-D score: -0.7±0.5 at day 50 -0.9±0.7 at day 218 | Mean change in HADS-A score: -0.5±0.7 at day 50 -1.0±1.5 at day 218  Mean change in HADS-D score: -0.2±0.5 at day 50 1.2±0.7 at day 218 | No difference |
| Patient global impression of change | DaSilva, 2012 [23] | 75% of patients had moderate improvement with partial remission of symptoms in the active group | 80% of patients had only slight improvement at the end of treatment | NR |
| Headache- and symptom-free days | Ahdab, 2019 [18] | 5.0* (weekly) at baseline  6.0* at week 1  5.7* at week 2  Significant increase in the number of headache-free  days at week 1 (0.62±0.11, p<0.001) but not at week 2: (0.45±0.14, p=0.006) | 5.4* (weekly) at baseline  5.7* at week 1  5.3* at week 2  No significant decrease compared with baseline | NR |
| Migraine-specific Quality-of-life questionnaire | De Icco, 2021 [24] | Role restrictive: 52.8±10.4 points at baseline, 61.1±13.5 at month 1  Role preventive: 62.9±12.0 points at baseline, 76.7±8.4 at month 1  Emotional: 61.0±11.4 points at baseline, 70.9±21.5 at month 1 | Role restrictive: 41.8±9.9 points at baseline, 52.0±15.6 at month 1  Role preventive: 57.5±16.6 points at baseline, 59.9±18.6 at month 1  Emotional: 55.5±14.6 points at baseline, 71.5±17.8 at month 1 | Differences in role restrictive (p=0.048), role preventive p=0.049), but not in emotional domain (p=0.684) |
| Headache Impact Test | Andrade, 2017 [19] | Median score at baseline:  61* (M1) 61* (DLPFC)  Median score immediately after tDCS: 52* (M1) 43* (DLPFC)  Significant decrease in score after treatment in both groups | Median score at baseline: 62*  Median score at follow-up: 60*  No significant decrease | NR |
|  | De Icco, 2021 [24] | 65.30±3.36 at baseline, 58.8±5.86 at month 1 | 66.6±3.3 at baseline, 65.6±4.3 at month 1 | p=0.006 |
|  | Grazzi, 2020 [25] | Score change: -4.4±9.1 (anodal) -4.7±8.8 (cathodal) | Score change: -3.6±6.9 | No difference |
| Migraine Disability Assessment Scale | De Icco, 2021 [24] | 71.3±36.5 at baseline, 63.8±36.9 at month 1 | 76.8±53.0 at baseline 78.4±35.8 at month 1 | p=0.561 |
|  | Grazzi, 2020 [25] | Score change:  -45.4±48.4 (anodal) -27.8±67.5 (cathodal) | Score change: -17.1±46.1 | No difference |
|  | Pohl, 2020 [27] | Score change:  5.9±4.1 at day 50 -1.8±5.9 at day 218 | Score change:  -7.7±4.6 at day 50 -3.7±6.3 at day 218 | No difference |
| 36-Item Short Form Health Survey | Andrade, 2017 [19] | Median at baseline:  63.9 (M1) 62.5 (DLPFC)  Median immediately after tDCS: 77.3 (M1) 86.9 (DLPFC) | Median at baseline: 63.2  Median at follow-up: 65.9 | NR |
|  | De Icco, 2021 [24] | Significant improvement in the overall population only in the sub-domain related to “role limitation due to physical health” (F_1,18_=5.099; p=0.037) | - | Significant improvement of “role limitation due to physical health” domain only in tDCS group (p=0.030) at post-hoc analysis |
|  | Wickmann, 2015 [30] | Improvement in several domains | Improvement in several domains | Difference only in the “social interactions” domain in favor of active group (p=0.05) |

*Values retrieved from graphs

BDI indicates Beck Depression Inventory; DLPFC, dorsolateral prefrontal cortex; HADS, Hospital Anxiety and Depression Scale; M1, primary motor cortex; STAIS, State-Trait Anxiety Inventory – State; STAIT, State-Trait Anxiety Inventory – Trait; tDCS, transcranial direct current stimulation; VAS, Visual Analog Scale
