# Supplementary material for: Which is the best transcranial direct current stimulation protocol for migraine prevention? A systematic review and critical appraisal of randomized controlled trials
Source: J Headache Pain. 2021 Nov 27;22(1):144. doi: 10.1186/s10194-021-01361-0 (PMC8903540; doi:10.1186/s10194-021-01361-0)
Supplement: Supplementary file 2 — Additional file 2. Adverse events in the included trials. [file 10194_2021_1361_MOESM2_ESM.docx]

Adverse events in the included randomized controlled trials

| **Study** | **Active group – during procedure** | **Active group – after procedure** | **Sham group – during procedure** | **Sham group – after procedure** |
| --- | --- | --- | --- | --- |
| Ahdab, 2019 [18] | 14.0% headache 6.3% pain 4.8% fatigue  4.8% disturbed vision 4.0% disturbed concentration 1.6% nervousness | 14.0% headache 3.2% pain 2.4% fatigue  2.4% disturbed vision 4.0% disturbed concentration 1.6% nervousness | 7.0% headache 3.2% pain 3.2% fatigue  1.6% disturbed vision 1.6% disturbed concentration 0.8% nervousness | 7.0% headache 2.4% pain 1.6% fatigue  0.8% disturbed vision 0.8% disturbed concentration |
| Andrade, 2017 [19] | Dizziness, tingling, headache, burning, sleepiness reported by >10% of patients | NR | Dizziness, tingling, headache, burning reported by >10% of patients | NR |
| Antal, 2011 [20] | 78.1% tingling  21.3% itching 14.3% fatigue | 7.1% tiredness 21.3% headache | 71.0% tingling  28.4% itching  28.4% fatigue | 42.6% tiredness  35.5% headache |
| Auvichapayat, 2012 [21] | 10.0% (n=2) headache  5.0% (n=1) first-degree burn (healed within five days)  5.0% (n=) drowsiness and rash under electrode (disappeared within two hours)  5.0% (n=1) headache, decreased appetite, rash | NR | 5.9% (n=1) rash under electrode  5.9% (n=1) itching  5.9% (n=1) headache, decreased appetite  5.9% (n=1) headache, dizziness | NR |
| Dalla Volta, 2020 [22] | None | None | None | None |
| DaSilva, 2012 [23] | 14.0% (n=7) headache  10.0% (n=5) neck pain  8.0% (n=4) tingling  4.0% (n=2) skin redness  2.0% (n=1) sleepiness  2.0% (n=1) scalp pain | NR | 27.8% (n=5) headache  11.1% (n=2) neck pain  11.1% (n=2) tingling  22.2% (n=4) skin redness  22.2% (n=4) sleepiness  5.6% (n=1) scalp pain | NR |
| De Icco, 2021 [24] | NR | NR | NR | NR |
| Grazzi, 2020 [25] | None | None | None | None |
| Mansour, 2019 [26] | NR | NR | NR | NR |
| Pohl, 2020 [27] | 54.5% (n=6) tingling  54.5% (n=6) fatigue  36.4% (n=4) pain  27.3% (n=3) nausea  9.1% (n=1) migraine during stimulation  9.1% (n=1) temporary pain in the right half of face | NR | 66.7% (n=8) tingling  33.3% (n=4) fatigue  41.7% (n=5) pain  8.3% (n=1) nausea  8.3% (n=1) migraine in close temporal relationship with procedure  8.3% (n=1) pain in right ear  8.3% (n=1) depressed mood | NR |
| Rahimi, 2020 [28] | 17.8% in primary motor cortex stimulation  8.9% in somatosensory cortex stimulation  (tingling, itching, or burning) | 6.7% in primary motor cortex stimulation  8.9% in somatosensory cortex stimulation  (mild, short-lived headache) | 20% (tingling, itching, or burning) | None |
| Rocha, 2015 [29] | 80.0% (n=8) tingling  70.0% (n=7) itching  60.0% (n=6) headache  60.0% (n=6) skin redness  50.0% (n=5) burning sensation  40.0% (n=4) sleepiness  20.0% (n=2) neck pain  20.0% (n=2) scalp pain  20.0% (n=2) other events | NR | 80.0% (n=4) tingling  60.0% (n=3) itching  40.0% (n=2) headache  60.0% (n=3) burning sensation  60.0% (n=3) sleepiness  40.0% (n=2) neck pain  20.0% (n=1) other events | NR |
| Wickmann, 2015 [30] | 83.3% (n=20) tingling  70.8% (n=17) itching  20.8% (n=5) burning  12.5% (n=3) headache  12.5% (n=3) unpleasantness  4.2% (n=1) changes in visual perception  4.2% (n=1) changes in concentration abilities | 45.8% (n=11) itching  33.3% (n=8) tingling  16.7% (n=4) headache  12.5% (n=3) sensation of cold  4.2% (n=1) burning  25.0% (n=6) other | 23.1% (n=6) pain  53.8% (n=14) tingling  38.5% (n=10) itching  26.9% (n=7) burning  15.4% (n=4) fatigue  7.7% (n=2) nervousness  3.8% (n=1) headache  3.8% (n=1) changes in visual perception  23.1% (n=6) unpleasantness  19.2% (n=5) changes in concentration abilities | 19.3% (n=5) fatigue  15.4% (n=4) itching  15.4% (n=4) burning  7.7% (n=2) pain  7.7% (n=2) tingling  7.7% (n=2) changes in concentration abilities  7.7% (n=2) headache  7.7% (n=2) nausea  7.7% (n=2) sensation of cold  3.8% (n=1) nervousness  3.8% (n=1) emesis  3.8% (n=1) increased mood  3.8% (n=1) anxiety  15.4% (n=4) other |

DLPFC indicates dorsolateral prefrontal cortex; M1, primary motor cortex; NR, not reported
